# Comparing the frequency of common genetic variants and haplotypes between carriers and non-carriers of *BRCA1 *and *BRCA2 *deleterious mutations in Australian women diagnosed with breast cancer before 40 years of age

**DOI:** 10.1186/1471-2407-10-466

**Published:** 2010-09-01

**Authors:** Lidija Turkovic, Lyle C Gurrin, Melanie Bahlo, Gillian S Dite, Melissa C Southey, John L Hopper

**Affiliations:** 1 Centre for Molecular, Environmental, Genetic and Analytic Epidemiology, School of Population Health, The University of Melbourne, Victoria 3010, Australia; 2 Walter and Eliza Hall Institute for Medical Research, 1G Royal Parade, Victoria 3052, Australia; 3 Department of Pathology, The University of Melbourne, Victoria 3010, Australia

## Abstract

**Background:**

*BRCA1 *and *BRCA2 *mutations are found in a proportion of families with multiple early-onset breast cancers. There are a large number of different deleterious mutations in both genes, none of which would be detectable using standard genetic association studies. Single common variants and haplotypes of common variants may capture groups of deleterious mutations since some low prevalence haplotypes of common variants occur more frequently among chromosomes that carry rare, deleterious mutations than chromosomes that do not.

**Methods:**

DNA sequence data for *BRCA1 *and *BRCA2 *was obtained from 571 participants from the Australian Breast Cancer Family Study. Genetic variants were classified as either deleterious mutations or common genetic variants. Variants tagging common polymorphisms were selected and haplotypes resolved using Haploview. Their frequency was compared to those with and without deleterious mutations using a permutation test.

**Results:**

A common genetic variant in *BRCA1 *(3232A > G) was found to be over-represented in deleterious mutation carriers (p = 0.05), whereas a common genetic variant in *BRCA2 *(1342A > C) occurred less frequently in deleterious mutation carriers (p = 0.04). All four of the common *BRCA1 *variants used to form haplotypes occurred more frequently in the deleterious mutation carriers when compared to the non-carriers, but there was no evidence of a difference in the distributions between the two groups (p = 0.34). In *BRCA2*, all four common variants were found to occur less frequently in the deleterious mutation carriers when compared to non-carriers, but the evidence for difference in the distribution between the two groups was weak (p = 0.16). Several less common haplotypes of common *BRCA1 *variants were found to be over-represented among deleterious mutation carriers but there was no evidence for this at the population level. In *BRCA2*, only the most common haplotype was found to occur more frequently in deleterious mutation carriers, with again no evidence at the population level.

**Conclusions:**

We observed differences in the frequency of common genetic variants of the *BRCA1 *and *BRCA2 *and their haplotypes between early-onset breast cancer cases who did and did not carry deleterious mutations in these genes. Although our data provide only weak evidence for a difference in frequencies at the population level, the number of deleterious mutation carriers was low and the results may yet be substantiated in a larger study using pooled data.

## Background

Mutations in *BRCA1 *and *BRCA2 *are found in a proportion of multiple case breast cancer families. The particular mutations that are present differ from family to family indicating the marked allelic heterogeneity of these genes. The only viable methods for the identification of mutations in genes prone to such variation are sequencing or extensive DNA screening techniques. Mutation screening of these genes has become widespread despite the costs involved. In fact, 890 and 975 non-protein truncating mutations have been identified in *BRCA1 *and *BRCA2 *respectively, making it difficult to identify causal mutations due to the large number of variants. Many such variants may appear deleterious but may nevertheless only be associated with disease because they are close to a causal mutation and not deleterious in their own right. An appealing strategy is therefore to avoid the large number of comparisons required to test each variant separately, but to instead use methods based on haplotypes, which are combinations of genetic variants or *alleles *(typically common polymorphisms) inherited together or *in phase *from a single parent. They result from the phenomenon of *linkage disequilibrium *(LD), where alleles at closely spaced markers do not segregate independently. Haplotypes can capture more information across genomic regions of interest on the human genome than is available by examining single genetic markers one at a time [1] however the generation of haplotypes is difficult, usually requiring intensive laboratory efforts or the collection and genotyping of several closely related relatives to infer phase. An alternative is to infer phase from genotype data using the statistical techniques that have been developed rapidly in the last few years, which is the approach adopted in this report.

Haplotypes are expected to play an important role in the fine mapping of complex diseases since disease-affected individuals with common haplotypes may have recent shared ancestry of chromosomal segments that harbour disease-causing variants [2]. By identifying "disease haplotypes" that are the hallmarks of deleterious *BRCA1 *and *BRCA2 *mutations, it may be possible to identify carriers implicitly rather than by screening of the entire gene. Some early work in this area has suggested that one haplotype in *BRCA1 *is over-represented in individuals carrying deleterious mutations [3] while another haplotype was associated with a 20% increased risk in breast cancer [4]. There is evidence that this scenario is particularly useful for common polymorphisms of low penetrance, where results show that an association can be detected via haplotype methods using single nucleotide polymorphisms surrounding the functional allele even if the functional allele is not typed [5]. We explored extending this approach to detecting rare deleterious mutations, which are likely to have arisen more recently and occur on extended, less common haplotypes.

## Methods

### Study Methods

The Australian Breast Cancer Family Study (ABCFS) [6,7] is a population based case-control-family study carried out in the metropolitan areas of Melbourne and Sydney between years 1992 and 1999. Potential case patients were identified through the Victorian and New South Wales cancer registries. Women were eligible if they were aged between 18 and 59 and had been diagnosed with histologically confirmed first primary cancer of the breast, irrespective of family history of breast cancer. Controls were women with no previous breast cancer, randomly selected from the electoral roll for which registration (and voting) is compulsory in Australia. Letters to cases were sent to both the attending doctor and patient inviting them to participate. Participation involved completing an interviewer administered risk factor questionnaire and giving a blood sample. A detailed family history was recorded for all first-degree and second-degree relatives, with verification sought for all reports of cancers in other family members. All patients provided written informed consent for participation in all aspects of the study. Sampling of cases was stratified by the age of onset, with over half being less than 40 years old. The majority of proband participants in the ABCFS are white women with northern or southern European ancestry. The protocols for ABCFS have been approved by the Human Research Ethics Committee of University of Melbourne. Details of recruitment strategy, participation, and data collection methods have previously been described [6].

### Molecular methods

The coding and exon flanking sequences of *BRCA1 *and *BRCA2 *were screened as described in [7]. These analyses were performed by Myriad Genetic Laboratories, Inc. using full sequence analyses (BRC-Analysis), in house DNA sequencing and 2 D gel electrophoresis, which are fully described in [8,9].

The criteria for defining deleterious mutations were those used by the Breast Information Core (BIC; http://research.nhgri.nih.gov/bic/ and Myriad Genetic Laboratories, Inc. and are described in more detail in [9].

### Statistical methods

A smaller set of haplotype tagging SNPs (htSNP) was identified to explain the diversity of common haplotypes [10] using the Haploview software [11]. Haploview was also used to calculate minor allele frequencies for *BRCA1 *and *BRCA2 *from the data. Haploview uses a two marker EM algorithm (ignoring missing data) to estimate the maximum-likelihood values of the four gamete frequencies, from which the calculations for the LD summary statistics D' **(**Lewontin's normalized coefficient D' [12]), LOD (likelihood ratio test statistic) and *r*^2 ^(paired correlation) are derived. Haplotype phase and an estimate of the population frequency distribution of the haplotypes are inferred using a standard EM algorithm with a partition-ligation approach for blocks with greater than 10 markers. The standard chi-squared statistics on 1 degree of freedom and a corresponding p-value to test the assumption of Hardy-Weinberg Equilibrium (HWE) values were also generated for each variant using Haploview.

To validate the calculations in Haploview, haplotypes of tag SNPs were also generated using the Bayesian simulation procedures incorporated in the PHASE software [13,14], which implements a Markov chain Monte Carlo (MCMC) algorithm. One advantage of this alternative software is that it can be used to generate standard errors for estimates of the population haplotype frequencies. We compared those standard errors to these generated from the sampling variability of a proportion using the binomial distributions and found that they were undistinguishable so we present the latter values based on the Haploview output only.

Individuals were classified into two groups according to whether their DNA specimen results showed that they had at least one deleterious mutation in *BRCA1 *and *BRCA2 *or not. Estimated frequencies of haplotypes as well as individual htSNP were compared between individuals carrying and not carrying deleterious mutations using the permutation test (with 10000 permutations) for association between SNP and case/control status that is implemented in Haploview. Haplotype distributions were also compared between groups with and without deleterious mutations by (i) using the permutation p-value for each single haplotype to generate a chi-squared statistic on 1 degree of freedom and then (ii) summing the chi-squared statistics to generate a combined statistics which was then compared to the chi-squared distribution with the relevant number of degrees of freedom to generate a p-value. These p-values were compared to the p-value generate by PHASE for comparing the full haplotype distribution. We found that in all cases these procedures gave the same "omnibus" p-value to two decimal places, which provides an empirical validation of the procedure used in PHASE.

## Results

Data was available on 680 participants in regard to *BRCA1 *and 245 participants for *BRCA2 *(table 1).

**Table 1:** Breast cancer cases with DNA sequence data and those carrying deleterious mutations by age group.

Age	*BRCA1 *total with sequenced data	*BRCA1 * deleterious mutation number	*BRCA2 *total with sequenced data	*BRCA2 * deleterious mutation number
<40	392	13	179	11
40-49	102	4	34	0
50-59	186	1	32	0
Total	680	18	245	11

Analyses was restricted to the 392 (*BRCA1*) and 179 (*BRCA2*) population-based individuals diagnosed with a first primary invasive breast cancer before 40 years of age for whom *BRCA1 *and *BRCA2 *had been sequenced. Some sequencing was performed for cases in other age groups but the sample sizes were small and not sufficient to warrant separate analysis. The total number of variants found in the coding regions of *BRCA1 *and *BRCA2 *gene in cases under 40 were 22 (table 2) and 15 (table 3) respectively. Each of the deleterious mutations identified in our sample appeared only once, with the exception of 2800 del AAG which occurred once in each of two study participants.

**Table 2:** Observed *BRCA1 *variants in breast cancer cases diagnosed before age 40 in the ABCFS.

Variant	rs number	Codon	MAF	HWE	Deleterious mutation?
188 del 11		C24X	0.001	1.000	Yes
189 del 11			0.003	1.000	Yes
546 G > T		E143X	0.001	1.000	Yes
1186 A > G	rs1799950	Q356R	0.056	0.557	No
1876 del C			0.001	1.000	Yes
2196 G > A	rs4986850	D693N	0.060	0.461	No
2201 C > T	rs1799949	S694S	0.227	0.000	No
2430 T > C	rs16940	L771L	0.218	0.000	No
2594 del C			0.001	1.000	Yes
2731 C > T	rs799917	P871L	0.231	0.000	No
2800 del AAG			0.001	1.000	Yes
3232 A > G	rs16941	E1038G	0.195	0.000	No
3415 del C			0.001	1.000	Yes
3667 A > G	rs16942	K1183R	0.231	0.000	No
3875 del GTCT			0.001	1.000	Yes
3888 del GAG			0.001	1.000	Yes
4184 del TCAA			0.003	1.000	Yes
4427 T > C	rs1060915	S1436S	0.231	0.000	No
4446 C > T	rs41293455	R1443X	0.001	1.000	Yes
4808 C > G		Y1563X	0.001	1.000	Yes
4956 A > G	rs1799966	S1613G	0.237	0.000	No
5382 ins C			0.003	1.000	Yes

**Table 3:** Observed *BRCA2 *variants in breast cancer cases diagnosed before age 40 in the ABCFS.

Variant	rs number	Codon	MAF	HWE	Deleterious mutation?
478 C > T		Q84X	0.003	1.000	Yes
1342 A > C	rs6004238	N372H	0.318	0.000	No
3642 A > G	rs1801406	K1132K	0.131	0.033	No
4035 T > C	rs543304	V1269V	0.117	0.038	No
4075 del GT			0.003	1.000	Yes
4856 del A			0.003	1.000	Yes
5638 del GT			0.003	1.000	Yes
5803 del ATTA			0.003	1.000	Yes
6174 del T			0.003	1.000	Yes
6503 del TT			0.006	1.000	Yes
7405 ins A			0.003	1.000	Yes
7470 A > G	rs1799955	S2414S	0.103	0.050	No
9097 C > T		Q2957X	0.003	1.000	Yes
9132 del C			0.003	1.000	Yes
9504 T > G		T3092X	0.003	1.000	Yes

Tables 4 and 5 list the details of the variants selected as tagSNPs by Haploview's Tagger program for *BRCA1 *and *BRCA2 *respectively. These tables display results of comparing the allele frequencies of these tagSNPs between individuals carrying and not carrying deleterious mutations. In both *BRCA1 *and *BRCA2 *there was some evidence that the tagSNPs allele frequency differed according to deleterious mutation status. Genotype frequencies for some variants generate small p-value for the test of HWE, most likely due to the fact that our analysis is restricted to breast cancer cases under age 40 years. The *BRCA1 *variant 3232A > G was found to occur more frequently in deleterious mutation carriers (p = 0.047) while *BRCA2 *variant 1342A > C was found to occur less frequently in deleterious mutation carriers (p = 0.043).

**Table 4:** Comparison of individual *BRCA1 *variant frequencies

Variant	rs number	Codon Change	Deleterious mutation non- carriers	Deleterious mutation carriers	Chi square (Permutation)	P value (Permutation)
1186 A > G	rs1799950	Q356R	0.054	0.107	0.180	0.671
2196 G > A	rs4986850	D693N	0.060	0.071	0.000	1.000
3232 A > G	rs16941	E1038G	0.189	0.357	3.945	0.047
4427 T > C	rs1060915	S1436S	0.228	0.321	0.146	0.702
Overall					4.541	0.338

**Table 5:** Comparison of individual *BRCA2 *variant frequencies

Variant	rs number	Codon Change	Deleterious mutation non-carriers	Deleterious mutation carriers	Chi square (Permutation)	P value (Permutation)
1342 A > C	rs6004238	N372H	0.338	0.042	4.095	0.043
3642 A > G	rs1801406	K1132K	0.141	0.000	1.392	0.238
4035 T > C	rs543304	V1269V	0.123	0.042	0.215	0.643
7470 A > G	rs1799955	S2414S	0.111	0.000	0.845	0.358
Overall					6.547	0.162

All four common *BRCA1 *variants used to form haplotypes occur more frequently in the deleterious mutation carriers when compared to the non-carrier group, but there was no evidence of a difference in the distribution between the two groups (p = 0.34). The opposite was true for *BRCA2*, where all four common variants were found to occur less frequently in the deleterious mutation carriers group when compared to non-carriers, but the evidence for difference in the distribution between the two groups was weak (p = 0.16).

When comparing haplotype frequencies between the two groups (tables 6 and 7), there was very weak evidence that haplotype AGGT in *BRCA1 *was over-represented in individuals carrying deleterious mutations (p = 0.151). Overall, there was no evidence of difference between haplotype distributions between deleterious mutation carriers and non-carriers in *BRCA1 *(with 6.d.f., p = 0.717).The most common haplotype in *BRCA2*, AATA, was also found to occur slightly more frequently in deleterious mutation carriers (p = 0.158). Again, there was no evidence of difference in *BRCA2 *haplotype distributions between the two groups (with 6.d.f., p = 0.851).

**Table 6:** Comparison of individual *BRCA1 *haplotype frequencies

Haplotype	Deleterious mutation noncarriers (standard error)	Deleterious mutation carriers (standard error)	All (standard error)	Chi square (Permutation)	P value (Permutation)
AATA	0.659 (0.025)	0.958 (0.011)	0.679 (0.025)	1.993	0.158
AGTG	0.003 (0.003)	0.000 (0.000)	0.003 (0.003)	0.000	1.000
CATA	0.078 (0.014)	0.000 (0.000)	0.073 (0.014)	0.205	0.651
CACA	0.119 (0.017)	0.042 (0.011)	0.114 (0.017)	0.044	0.833
CACG	0.003 (0.003)	0.000 (0.000)	0.003 (0.003)	0.000	1.000
CGTA	0.033 (0.009)	0.000 (0.000)	0.031 (0.009)	0.012	0.911
CGTG	0.104 (0.016)	0.000 (0.000)	0.097 (0.016)	0.394	0.530
Overall				2.648	0.851

**Table 7:** Comparison of individual *BRCA2 *haplotype frequencies

Haplotype	Deleterious mutation noncarriers (standard error)	Deleterious mutation carriers (standard error)	All (standard error)	Chi square (Permutation)	P value (Permutation)
AGAT	0.691 (0.017)	0.527 (0.018)	0.685 (0.017)	0.953	0.329
AGAC	0.043 (0.002)	0.000 (0.000)	0.041 (0.007)	0.046	0.830
AGGT	0.004 (0.002)	0.036 (0.007)	0.005 (0.003)	2.062	0.151
AGGC	0.148 (0.003)	0.258 (0.016)	0.152 (0.013)	0.513	0.474
AAAT	0.023 (0.005)	0.008 (0.003)	0.022 (0.005)	0.000	1.000
AAGC	0.037 (0.007)	0.063 (0.009)	0.038 (0.007)	0.002	0.965
GGAT	0.054 (0.008)	0.107 (0.011)	0.056 (0.008)	0.132	0.716
Overall				3.708	0.717

## Discussion

In this paper we used *BRCA1 *and *BRCA2 *sequence data from Australian breast cancer cases less than 40 years of age at the time of diagnosis to classify individuals according to their deleterious mutation status, and resolved haplotypes of common polymorphisms separately in the groups that did and did not carry deleterious mutations.

We found weak evidence that one haplotype of *BRCA1 *variants is over-represented in carriers of deleterious mutations. This haplotype contains the minor allele for 3232A > G variant which we found to be over-represented among deleterious mutation carriers. Other haplotypes containing the minor allele "G" also occurred more frequently in deleterious mutation group.

In *BRCA2 *we found evidence that the population frequency of the most common haplotype in individuals carrying deleterious mutations was greater than 95%, when the corresponding frequency in those without deleterious mutations was only 65%. Individuals without this haplotype are unlikely to carry deleterious mutations but the predictive power of this haplotype for deleterious mutations is low since it occurs very frequently in those with no deleterious mutations (namely the vast majority of the population).

The sample size of the deleterious mutation group was small for both genes, with only 13 and 11 individuals carrying deleterious mutations for *BRCA1 *and *BRCA2 *respectively. The power to detect differences in haplotype frequencies is therefore quite low, which might explain some of the high p-values obtained from permutation testing.

Our selection of tagSNPs for *BRCA1 *gene has one in common with tagSNPs selection of Osorio et al. [3] (where they have used 4427T- > C as a tagSNP) and Cox et al. [4] (Q356R as a tagSNP). In Osorio et al. their class II haplotype occurs more frequently among *BRCA1 *mutation carriers. This haplotype is essentially characterized by the 4427C- > T variant allele which was used as a tagSNP in our study. We found that minor allele occurred more frequently in deleterious mutation carriers compared to non-carriers (32% vs 23%) but the evidence for this at the population level was weak p = 0.25. Cox et al. found slight increase in risk of breast cancer with the Q356R polymorphism, contradicting an earlier result showing an inverse association [15]. We found that Q356R occurred more frequently among deleterious mutation carriers but again the evidence at the population level was weak.

There have been several case control studies seeking *BRCA1 *and *BRCA2 *variants associated with an increased risk of breast cancer. Freedman at al. [16] investigated if common *BRCA2 *variants contribute to the more common forms of breast cancer in a large multiethnic cohort. Twenty one tagging SNPs were selected to predict common *BRCA2 *haplotypes. A number of haplotypes were found to be associated with increased risk of breast cancer, all of which could be attributed to a single marker (intron 24: rs206340) that was not selected as a tag SNP for analysis in our study. Freedman at al. [17] repeated similar analysis on *BRCA1 *gene. Specifically, they have used 28 variants to define patterns of common variation (5 in common with variants used in our study: Q356R, P871L, K1183R, S1613G and E1038G). They found no evidence for significant association between common variation in *BRCA1 *and risk of breast cancer.

The suggestive associations that we have observed do not imply a physical association on the same chromosome (as would be the case if the rare, deleterious mutation was in *cis *phase with a haplotype consisting of, for example, the minor alleles of several common variants) or a functional association (as might be the case even if the rare, deleterious mutation was in *trans *phase with a common variant haplotype, since it may still act to modify the penetrance of the disease causing variant). Establishing the phase of rare, deleterious mutations and the common variants we used to define haplotypes for both *BRCA1 *and *BRCA2 *would require either a much larger sample size than was available for this study, genetic data from extended pedigrees or expensive laboratory investigation.

## Conclusions

We found some evidence that a single common *BRCA1 *variant occurs more frequently in deleterious mutation carriers. All four common variants used to form *BRCA1 *haplotypes are over represented in deleterious mutation carriers so the frequency of less common haplotypes is also greater in this group but there is no evidence for this at the population level. In *BRCA2*, there is some evidence that a single common variant is under-represented in deleterious mutation carriers so the most common *BRCA2 *haplotype occurs more frequently in this group. All four common variants used to form *BRCA2 *haplotypes occur less frequently in deleterious mutation carriers. We found no evidence of difference in haplotype distributions between the two groups in both genes, which concords with previous research. These findings are unlikely to have implications for screening at the population level for *BRCA1 *or *BRCA2*.

## Abbreviations

LD: Linkage Disequilibrium; ABCFS: Australian Breast Cancer Family Study; HWE: Hardy-Weinberg Equilibrium; MCMC: Markov Chain Monte Carlo.

## Competing interests

The authors declare that they have no competing interests.

## Authors' contributions

JLH conceived the idea of the ABCFS and co-designed the current study. LCG assisted with statistical analysis and writing the manuscript. GSD designed and maintained the database for ABCFS, conceptualized the structure of data extracts for the current study and assisted with data formatting for current analysis. MCS supervised and in some cases performed the sequencing and genotyping of variants used in this study and is responsible for the biospecimen repository from which samples were drawn. MB co-designed the current study, advised on haplotype analysis and statistical analysis of genetic variants and did critical edits of the statistical methods section of the manuscript. LT collated data into required format for genetic association analysis, performed analysis and wrote the draft of the paper. All authors read and approved the final manuscript.

## Pre-publication history

The pre-publication history for this paper can be accessed here:

http://www.biomedcentral.com/1471-2407/10/466/prepub
